# Sex‐ and site‐specific associations of circulating lipocalin 2 and incident colorectal cancer: Results from the EPIC cohort

**DOI:** 10.1002/ijc.35205

**Published:** 2024-11-07

**Authors:** Robin Reichmann, Katharina Nimptsch, Tobias Pischon, Marc J. Gunter, Mazda Jenab, Anne Kirstine Eriksen, Anne Tjonneland, Jürgen Janke, Verena Katzke, Rudolf Kaaks, Matthias B. Schulze, Fabian Eichelmann, Giovanna Masala, Sabina Sieri, Fabrizio Pasanisi, Rosario Tumino, Maria Teresa Giraudo, Joseph Rothwell, Gianluca Severi, Paula Jakszyn, Maria Jose Sanchez‐Perez, Pilar Amiano, Sandra M. Colorado‐Yohar, Marcela Guevara, Bethany van Guelpen, Elom K. Aglago, Alicia K. Heath, Karl Smith‐Byrne, Elisabete Weiderpass, Krasimira Aleksandrova

**Affiliations:** ^1^ Biomarkers and Metabolism Research Group, Department of Epidemiological Methods and Etiological Research Leibniz Institute for Prevention Research and Epidemiology Bremen Germany; ^2^ Molecular Epidemiology Research Group Max‐Delbrueck‐Center for Molecular Medicine in the Helmholtz Association (MDC) Berlin Germany; ^3^ Biobank Technology Platform Max‐Delbrueck‐Center for Molecular Medicine in the Helmholtz Association (MDC) Berlin Germany; ^4^ Core Facility Biobank Berlin Institute of Health at Charité—Universitätsmedizin Berlin Berlin Germany; ^5^ Charité—Universitätsmedizin Berlin Corporate Member of Freie Universität Berlin and Humboldt‐Universität zu Berlin Berlin Germany; ^6^ International Agency for Research on Cancer World Health Organization Lyon France; ^7^ Department of Epidemiology and Biostatistics, School of Public Health Imperial College London London UK; ^8^ Diet, Cancer and Health Research Group Danish Cancer Institute, Danish Cancer Society Copenhagen Denmark; ^9^ Department of Public Health University of Copenhagen Copenhagen Denmark; ^10^ Department of Cancer Epidemiology German Cancer Research Center (DKFZ) Heidelberg Germany; ^11^ Department of Molecular Epidemiology German Institute of Human Nutrition Potsdam‐Rehbruecke Nuthetal Germany; ^12^ Institute of Nutritional Science University of Potsdam Nuthetal Germany; ^13^ German Center for Diabetes Research (DZD) Neuherberg Germany; ^14^ Prevention and Clinical Network Institute for the Study and Prevention of Cancer (ISPRO) Florenz Italy; ^15^ Epidemiology and Prevention Unit Fondazione IRCCS Instituto Nazionale dei Tumori Milan Italy; ^16^ Dipartimento di Medicina Clinica e Chirurgia Federico II University Naples Italy; ^17^ Hyblean Association for Epidemiological Research Associazione Iblea per la Ricerca Epidemiologica (A.I.R.E.–ONLUS) Ragusa Italy; ^18^ Department of Clinical and Biological Sciences University of Turin Turin Italy; ^19^ CESP—Univ. Paris‐Saclay, UVSQ, Inserm—"Exposome, heredity, cancer and health" Team The Centre for Research in Epidemiology and Population Health Villejuif France; ^20^ Department of Statistics, Computer Science, Applications “G. Parenti” (DISIA) University of Florence Florence Italy; ^21^ Unit of Nutrition and Cancer, Cancer Epidemiology Research Programme Catalan Institute of Oncology (ICO‐IDIBELL) Barcelona Spain; ^22^ Blanquerna School of Health Sciences Ramon Llull University Barcelona Spain; ^23^ CIBER of Epidemiology and Public Health (CIBERESP) Madrid Spain; ^24^ Public Health Research and Health Services Research Group Andalusian School of Public Health (EASP) Granada Andalucía Spain; ^25^ Epidemiology, Prevention and Control of Cancer Research Group Biosanitary Research Institute of Granada (ibs.Granada) Granada Spain; ^26^ Department of Preventive Medicine and Public Health University of Granada Granada Spain; ^27^ Sub Directorate for Public Health and Addictions of Gipuzkoa Ministry of Health of the Basque Government San Sebastian Spain; ^28^ Epidemiology of Chronic and Communicable Diseases Group Biodonostia Health Research Institute San Sebastian Spain; ^29^ Instituto de Salud Carlos III CIBER of Epidemiology and Public Health (CIBERESP) Madrid Spain; ^30^ Department of Epidemiology, Murcia Regional Health Council Instituto Murciano de Investigación Biosanitaria Murcia Spain; ^31^ Research Group on Demography and Health, National Faculty of Public Health University of Antioquia Medellin Colombia; ^32^ Epidemiology and Health Prevention Service Institute of Public Health and Labor of Navarre Pamplona Navarra Spain; ^33^ Epidemiology of Cancer and Other Chronic Diseases Research Group Healthcare Research Institute of Navarre (IdiSNA) Pamplona Spain; ^34^ Department of Radiation Sciences Umeå University Umeå Sweden; ^35^ Wallenberg Centre for Molecular Medicine Umeå University Umeå Sweden; ^36^ Cancer Epidemiology Unit, Nuffield Department of Population Health University of Oxford Oxford UK; ^37^ Faculty of Human and Health Sciences University of Bremen Bremen Germany

**Keywords:** colorectal cancer, EPIC, immunity, lipocalin 2, metabolism

## Abstract

Experimental research has uncovered lipocalin 2 (LCN2) as a novel biomarker implicated in the modulation of intestinal inflammation, metabolic homeostasis, and colon carcinogenesis. However, evidence from human research has been scant. We, therefore, explored the association of pre‐diagnostic circulating LCN2 concentrations with incident colorectal cancer (CRC) in a nested case–control study within the in the European Prospective Investigation into Cancer and Nutrition (EPIC) cohort. LCN2 was measured in 1267 incident CRC cases matched to 1267 controls using incidence density sampling. Conditional logistic regression was used to estimate incidence rate ratios (IRRs) and 95% confidence intervals (95% CIs) according to tumor subsite and sex. Weighted Cox proportional hazard regression was used to explore associations by adiposity status. In multivariable‐adjusted analyses, the IRR [95% CI] per doubling in LCN2 concentration was 1.16 [0.98–1.37] for CRC overall, 1.26 [1.00–1.59] for colon cancer, and 1.08 [0.85–1.38] for rectal cancer. The association for colon cancer was more pronounced in women (IRR [95% CI], 1.66 [1.20–2.30]) and for proximal colon cancer (IRR [95% CI], 1.96 [1.15–3.34]), whereas no association was seen in men and distal colon cancer. The association for colon cancer was positive in individuals with high waist circumference (hazard ratio [95% CI], 1.69 [1.52–1.88]) and inverse in individuals with low waist circumference (hazard ratio [95% CI], 0.86 [0.76–0.98], *P* interaction<0.01). Overall, these data suggest that pre‐diagnostic LCN2 concentrations were positively associated with colon cancer, particularly occurring in the proximal colon, in women and among individuals with abdominal adiposity.

## What's New?

Chronic inflammation can lead to cancer, particularly in the colon, and a glycoprotein associated with inflammation, lipocalin‐2 (LCN2), has been implicated in cancer progression. Here, using data from the European Prospective Investigation into Cancer and Nutrition cohort (EPIC), the authors show that higher levels of circulating LCN2 are associated with an increased risk of colon cancer in women. No association was seen in men. The association was particularly pronounced in people with higher waist circumference and for cancers arising in the proximal colon.

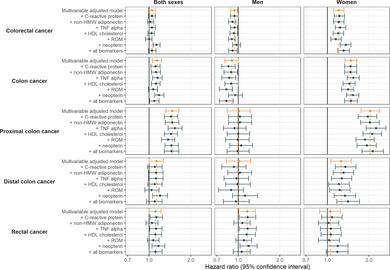

## 1 INTRODUCTION

Inadequate immune response to internal or external stimuli, prolonged inflammatory signaling, and failure in anti‐inflammatory mechanisms are known to predispose to a chronic pro‐inflammatory state and trigger tumorigenesis at various cancer sites.^1^ The colon may be especially susceptible to carcinogenesis due to the presence of microbial flora exposing its mucosa to persistent low‐grade inflammation.^2^ So far, several adipokines, chemokines, and acute‐phase reactants have been characterized in relation to colorectal cancer (CRC).^3^ Among the palette of newly established molecules, lipocalin 2 (LCN2) could represent an attractive molecular mediator linking chronic inflammation and CRC risk.^4^

LCN2 was originally identified as a 25‐kDa glycoprotein secreted from human immune cells such as neutrophils and macrophages highly regulated through onset of inflammation.^5^ LCN2 is abundantly expressed in adenomas and inflamed epithelia of the colon and its measurement reflects disease activity in inflammatory bowel diseases.^6^, ^7^, ^8^ LCN2 has been further described as an adipokine playing critical roles in the regulation of energy metabolism and insulin resistance^9^, ^10^, ^11^ and was implicated in cancer‐promoting processes including increased cell proliferation, angiogenesis, invasion, and metastasis.^12^, ^13^, ^14^ It has been shown to be overexpressed in colorectal neoplasms,^12^ and higher circulating LCN2 concentrations have been observed in CRC patients as compared to cancer‐free controls.^14^, ^15^, ^16^ Collectively, LCN2 has been suggested to exert multifaceted roles in the modulation of intestinal and metabolic inflammation, iron homeostasis,^17^, ^18^ as well as in colon cancer initiation and promotion.^13^, ^18^ Its potential role in the development of CRC, however, has not been explored in a prospective cohort study setting.

We, therefore, aimed to explore the association of pre‐diagnostic circulating concentrations of LCN2 with incident CRC and its subsites, by sex and adiposity status, in a nested case–control study within the European Prospective Investigation into Cancer and Nutrition (EPIC) cohort.

## 2 MATERIALS AND METHODS

### 2.1 Study population

The EPIC study is a prospective multicenter cohort study with around 521,468 participants from 23 study centers in 10 European countries, including Denmark, France, Germany, Greece, Italy, the Netherlands, Norway, Spain, Sweden, and the United Kingdom. Participants aged between 25 and 70 years were recruited between 1992 and 2000 predominantly from the general population. The study population and recruitment procedures have been described in detail elsewhere.^19^ Blood samples were collected at study baseline from 387,889 participants using standardized procedures. For most EPIC centers, half of the blood samples were stored locally, and half were transported to the central IARC repository to be stored in vapor phase of liquid nitrogen at −196°C.

### 2.2 Nested case–control study

Within the EPIC cohort, a nested case–control study was designed based on all incident CRC cases identified until December 2005. Cases were ascertained through record linkage of regional cancer registries (in Denmark, most centers in Italy, the Netherlands, Spain, Sweden, and the United Kingdom) or based on a combination of health insurance records, cancer‐ and pathology registries, as well as active follow‐up (in France, Germany, and Naples). CRC was defined as a combination of tumors in the colon (10th Revision of the International Classification of Diseases [ICD‐10] codes C18.0–C18.7), tumors that were overlapping or unspecified (C18.8–C18.9), and tumors of the rectum (C19–C20).^20^ Control participants were selected following an incidence density sampling approach. One control was selected for each case from a sample of those who were at risk at the time of diagnosis of the index case with available blood samples and matched (1:1) by recruitment center, sex, age at recruitment (±2 years), date of blood collection (±3 months), time of day of blood collection (±4 h), fasting status at blood collection (not fasting (<3 h), in‐between (3–6 h), fasting (>6 h)), and unknown, as well as (for most recruitment sites) menopausal status (premenopausal, postmenopausal, perimenopausal, or surgically postmenopausal) for women. Premenopausal women were matched on phases of menstrual cycles, and use of oral contraceptives, and postmenopausal women were matched on current hormone replacement therapy (HRT). The current analysis was based on a subsample of participants from all EPIC centers, except for Greece and Norway. LCN2 concentrations were measured in 1353 CRC cases and 1356 controls. After exclusion of all cases and controls of incompletely matched pairs (*N* = 175), the final study sample comprised 1267 first‐incident CRC cases and 1267 controls (Supplementary Figure S1).

### 2.3 Biomarker measurements

Serum LCN2 concentrations were measured using commercially available sandwich ELISA kit at the laboratory facilities of the manufacturer (BioVendor Laboratory Medicine, Inc.; Brno, Czech Republic). Measurements were performed according to the manufacturer's protocols (http://www.biovendor.com). The coefficients of variation ranged from 2.5 to 7.7 for the intra‐assay variation, and from 3.9 to 9.8 for the inter‐assay variation. The reported lower limit of detection was 0.02 ng/mL.^21^ In preliminary analyses, we assessed the reproducibility of LCN2 over a 4‐month period and observed relatively good stability with an intraclass correlation coefficient estimate of 0.64 (95% CI, 0.55–0.71).^21^ The measurements of biomarkers additionally included in the statistical analyses were described in detail elsewhere.^22^, ^23^, ^24^

### 2.4 Handling of missing data

In the analytical study sample, the data for selected covariates were partially incomplete (Table S1). Missing information was imputed using a nonparametric missing value imputation method based on random forest algorithms for mixed‐type data. A multivariate imputation model was generated using all available information on CRC status and matching factors using the corresponding *missForest* ^25^ package (version 1.4) for the statistical programming language R (version 4.1.1).

### 2.5 Statistical analyses

In descriptive analyses, baseline characteristics of the study participants were evaluated according to case–control status. Demographic, lifestyle, and anthropometric variables were further evaluated according to quartiles of LCN2 distribution in control participants. Spearman partial correlation coefficients, adjusted for age at study recruitment and sex, were estimated to assess the correlations between baseline LCN2 concentrations and additional inflammatory and metabolic markers measured in the control participants. Association of LCN2 with risk of CRC was evaluated using conditional logistic regression modeled continuously per doubling of LCN2 concentration and categorically according to quartiles of LCN2 concentrations in control participants. The nested case–control design and risk set sampling of control participants provided unbiased estimates of the corresponding incidence rate ratios (IRRs) in the underlying source population.^26^ In multivariable‐adjusted analyses, a priori chosen covariates were included as potential confounders beyond matching factors, including waist circumference, smoking status, physical activity, alcohol consumption, and daily intake of vegetables, fruits, red and processed meat, fish, and fiber. In CRC subsite‐specific analyses, the differences between LCN2 associations with CRC subsites were assessed with Lunn–McNeil competing risk analyses modeling site‐specific cancer outcomes as separate competing outcomes.^27^ In additional analyses, the associations were further adjusted for several biomarkers shown to be associated with CRC risk in previous research, including (high‐sensitivity) C‐reactive protein ((hs)CRP), non‐HMW (high molecular weight) adiponectin, tumor necrosis factor alpha (TNFα), high‐density lipoprotein (HDL) cholesterol, reactive oxygen metabolite (ROM), and neopterin. The shape of associations was evaluated using restricted cubic spline regression with three knots located at the 10th, 50th, and 90th percentiles of LCN2 distribution. The Wald test was used to assess the significance of non‐linear spline terms. The associations of LCN2 concentrations and CRC risk were assessed according to tumor subsite, sex, and waist circumference categories following predefined cut points based on the harmonized definition for metabolic syndrome for the European population.^28^ Statistical interaction was assessed by including multiplicative interaction terms and calculating *p*‐values using the Wald test, with *p* <.05 as a threshold of statistical significance. To optimize efficiency of subgroup analysis, an inverse‐probability weighting approach was applied to analyze the associations stratified by waist circumference.^29^ In this approach, accounting for the matching criteria, individual sampling probabilities of cases and controls of the study sample were calculated based on the original cohort data.^30^, ^31^ The inverse values of the calculated sampling probabilities were then used as sampling weights in weighted Cox proportional hazards regression. In sensitivity analyses, the analyses were repeated with the exclusion of (i) participants with less than 2 years of study follow‐up (*n*_cases_ = 231, *n*_controls_ = 231), (ii) participants with extreme LCN2 concentration levels (defined as below or above the 1st and 99th sex‐specific LCN2 percentile [*n*_cases_ = 53, *n*_controls_ = 53], and (iii)) participants with missing covariate data (*n*_cases_ = 77, *n*_controls_ = 77). We further conducted analyses restricted to (iv) postmenopausal women only (*n*_cases_ = 509, *n*_controls_ = 509). In addition, multivariable‐adjusted risk estimates were examined according to dichotomized LCN2 variable using 50 ng/mL, 30 ng/mL, and 25 ng/mL as predefined cut‐points. Statistical significance was assessed with 2‐sided *p‐*values and a significance level of 0.05. The sampling weights for the inverse probability weighting were calculated using the statistical programming language R (version 4.1.1, base only). Additional analyses were performed using Base SAS® software (version 9.3_M2) and SAS/STAT® software (version 12.1). All authors had access to the study data, and reviewed, and approved the final manuscript.

## 3 RESULTS

The median time from baseline to CRC diagnosis was 8.7 (interquartile range [IQR], 4.5–15.9) years. The median concentration of LCN2 was 24.1 (IQR, 19.7–30.3) ng/mL in men and 23.3 (IQR, 19.2–28.7) ng/mL in women (Figure S2a,b). Table 1 presents the descriptive characteristics of the study participants according to case and control status; Table 2 shows the descriptive characteristics of control participants according to LCN2 quartiles. Higher concentrations of LCN2 were associated with higher age at blood collection, higher waist circumference, and a higher prevalence of physical inactivity, but with a lower prevalence of never smoking and lower alcohol consumption. The proportion of postmenopausal women and those using oral contraceptives or HRT was lower in participants with high LCN2 concentrations. LCN2 was positively correlated with (hs)CRP, TNFα, ROM, as well as neopterin, whereas negatively correlated with non‐HMW adiponectin, and HDL cholesterol (Table S2).

**TABLE 1:** Descriptive characteristics of the study population by case control status.

Characteristics	Colorectal cancer	
	Cases (*N* = 1267)	Controls (*N* = 1267)
Age at blood collection^a^, median (IQR), years	58.7 (53.4–62.6)	58.6 (53.4–62.6)
Women^a^, %	51.9	51.9
Postmenopausal women^a^, %	38.7	39.5
Oral contraceptives/HRT in women^a^, %	8.6	8.7
BMI, median (IQR), kg/m^2^	26.5 (24.1–29.1)	26.0 (23.7–28.5)
Waist circumference, median (IQR), cm	90.0 (81.5–99.5)	88.0 (80.0–97.0)
Highest education, %		
None	4.7	4.4
Primary school completed	35.1	37.3
Technical/professional school	24.9	26.0
Secondary school	15.3	12.6
Longer education (incl. University degree)	17.4	17.7
Unspecified	2.6	2.1
Smoking status, %		
Never smoker	40.3	40.6
Former smoker	33.3	33.3
Current smoker	25.6	25.0
Unspecified	0.9	1.1
Physical activity, %		
Inactive	24.9	21.6
Moderately inactive	31.1	32.8
Moderately active	22.6	19.5
Active	20.4	24.7
Unspecified	1.0	1.4
Dietary intake, median (IQR), g/day		
Alcohol	8.6 (1.5–24.0)	7.9 (1.7–21.6)
Vegetables	156.5 (102.0–234.5)	157.5 (100.6–241.2)
Fruits	184.8 (100.4–289.3)	189.7 (105.8–312.2)
Red meat	48.3 (25.2–76.5)	47.3 (25.3–74.2)
Processed meat	25.7 (13.7–44.4)	25.1 (13.2–44.4)
Fish	28.2 (15.2–49.1)	29.4 (14.7–50.9)
Fiber	22.0 (17.6–27.4)	23.0 (18.0–27.8)

Abbreviations: BMI, body mass index; IQR, interquartile range.

^a^ Age, sex, menopausal status, and oral contraceptives/HRT use in women were among the matching criteria.

**TABLE 2:** Baseline characteristics among control participants (*N* = 1267), by quartiles of LCN2 concentrations.

Characteristics	LCN2 quartile categories (median [range])			
	1	2	3	4
	16.6 (7.7–<19.2 ng/mL)	21.5 (19.2–<23.6 ng/mL)	26.0 (23.6–<29.0 ng/mL)	34.4 (29.0–228.3 ng/mL)
*N* control participants	316	315	315	321
Age at blood collection, median (IQR), years	57.4 (52.5–61.6)	57.7 (52.8–62.2)	59.4 (54.9–62.8)	60.0 (54.1–64.0)
Women, %	56.3	54.3	52.1	44.9
Postmenopausal women, %	75.8	72.5	79.3	77.1
Oral contraceptives/HRT in women, %	12.0	10.5	7.6	4.7
BMI, median (IQR), kg/m^2^	26.3 (23.8–29.1)	26.2 (24.2–28.5)	26.1 (23.9–28.5)	25.7 (23.3–27.9)
Waist circumference, median (IQR), cm	87.5 (79.0–98.8)	87.3 (79.7–96.0)	89.7 (80.5–98.0)	89.0 (80.9–95.3)
Highest education, %				
None	5.7	4.4	2.5	5
Primary school completed	32.9	38.7	40.6	36.8
Technical/professional school	27.2	24.8	24.8	27.1
Secondary school	13.9	13.3	13.7	9.7
Longer education (incl. University degree)	19.3	17.8	16.5	17.1
Unspecified	0.9	1.0	1.9	4.4
Smoking status, %				
Never smoker	40.5	46.3	40	35.5
Former smoker	34.8	32.7	33	32.7
Current smoker	23.4	19.7	27	29.9
Unspecified	1.3	1.3	0.0	1.9
Physical activity, %				
Inactive	18.0	20.6	22.2	25.5
Moderately inactive	33.9	31.1	32.7	33.3
Moderately active	17.1	23.2	18.4	19.3
Active	30.1	23.5	24.8	20.6
Unspecified	0.9	1.6	1.9	1.2
Dietary intake, median (IQR), g/day				
Alcohol	10.3 (2.9–27.1)	8.7 (1.9–23.9)	7.0 (1.2–17.6)	6.2 (1.4–18.1)
Vegetables	153.7 (102.4–220.0)	162.0 (99.1–242.4)	157.4 (101.8–248.1)	161.4 (96.8–252.0)
Fruits	187.5 (98.5–302.0)	195.2 (107.3–330.1)	191.7 (114.1–330.2)	180.0 (100.9–287.8)
Red meat	46.9 (26.0–79.9)	48.5 (25.6–73.6)	48.3 (26.3–73.9)	45.7 (21.6–74.1)
Processed meat	26.6 (14.9–44.6)	25.2 (13.6–47.7)	23.0 (12.5–38.7)	24.2 (12.6–45.4)
Fish	31.4 (14.2–53.0)	28.8 (13.5–49.0)	29.6 (16.1–50.8)	28.2 (13.3–49.5)
Fiber	23.1 (17.9–28.3)	23.2 (18.2–27.6)	22.9 (18.2–28.6)	22.6 (17.7–27.3)
Biomarkers, median (IQR)				
C‐reactive protein, mg/L	2.4 (1.4–3.6)	2.6 (1.8–3.9)	2.7 (1.6–3.9)	3.5 (2.3–5.24)
Non‐HMW adiponectin, μg/mL	3.6 (2.9–4.0)	3.5 (3.0–4.1)	3.4 (2.9–4.0)	3.3 (2.7–3.8)
TNF alpha, pg./mL	2.0 (1.5–2.4)	2.1 (1.7–2.6)	2.2 (1.7–2.7)	2.6 (2.0–3.3)
HDL cholesterol, mmol/L	1.5 (1.3–1.7)	1.5 (1.3–1.7)	1.4 (1.2–1.6)	1.4 (1.2–1.5)
ROM, U/mL	390.0 (359.9–412.9)	386.0 (354.4–422.0)	390.0 (357.1–425.5)	400.0 (363.6–434.0)
Neopterin, nmol/L	16.9 (11.0–21.0)	16.5 (11.5–20.8)	17.6 (11.7–21.5)	19.7 (12.2–24.3)

Abbreviations: BMI, body mass index; HDL, high‐density lipoprotein; HMW, high molecular weight; IQR, interquartile range; LCN2, lipocalin 2; ROM, reactive oxygen metabolites; TNF, tumor necrosis factor.

**FIGURE 1:**
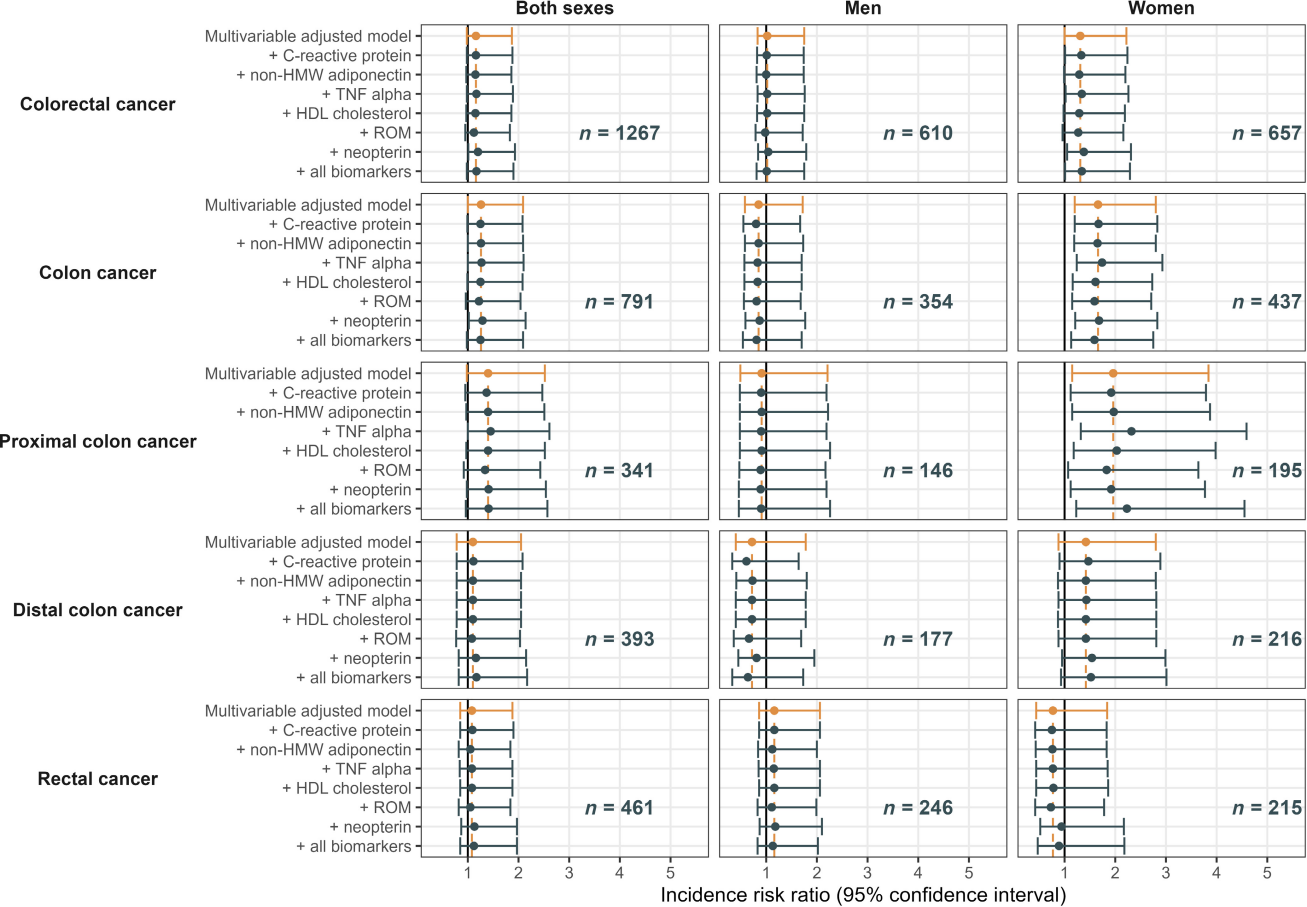
Incidence rate ratios (IRRs) and 95% confidence intervals (CIs) for the association of LCN2 with colorectal cancer and its subsites per doubling in LCN2 concentration after adjustment for additional biomarkers. Multivariable‐adjusted IRRs accounting for matching factors: Age, sex, study center, follow‐up time since blood collection, time of the day at blood collection and fasting status. Women were further matched by menopausal status and phase of menstrual cycle at blood collection; postmenopausal women were matched by hormone replacement therapy use. The model was further adjusted for smoking status, alcohol consumption, physical activity, fiber intake, consumption of fruits and vegetables, red and processed meat, fish and shellfish, and waist circumference. CI, confidence intervals; HDL, high‐density lipoprotein; HMW, high molecular weight; IRR, incidence rate ratios; LCN2, lipocalin 2; ROM, reactive oxygen metabolites.

Table 3 presents the IRRs and 95% CIs for the association of LCN2 with CRC and its subsites, overall and by sex. After adjustment for matching factors, waist circumference, smoking status, physical activity, alcohol consumption, daily intake of vegetables, fruits, red and processed meat, fish, and fiber, the adjusted IRR (95% CI) per log_2_ increase in LCN2 concentrations was 1.16 (0.98–1.37) for CRC overall, 1.26 (1.00–1.59) for colon cancer, and 1.08 (0.85–1.38) for rectal cancer. The association between LCN2 and colon cancer risk was found in women (adjusted IRR [95% CI], 1.66 [1.20–2.30]), but not in men (adjusted IRR [95% CI], 0.85 [0.58–1.22], *P* difference <.01). In women, the observed association between LCN2 and CRC was present for proximal colon cancer (adjusted IRR [95% CI], 1.96 [1.15–3.34]), whereas for distal colon cancer, the association did not reach statistical significance (adjusted IRR [95% CI], 1.42 [0.88–2.30]). In restricted cubic spline regression analyses, no deviation from log‐linearity was observed except for the associations in women with overall CRC and rectal cancer (Figure S3). Table S3 provides a more detailed overview of the associations according to LCN2 quartiles using the first quartile as reference. In analyses stratified by waist circumference categories, a positive association with colon cancer risk was observed in individuals with a waist circumference ≥94 cm for men and ≥80 cm for women (adjusted HR [95% CI], 1.69 [1.52–1.88]), whereas in individuals with a waist circumference <94 cm for men and <80 cm for women an inverse association was seen (adjusted HR [95% CI], 0.86 [0.76–0.98], *P*_interaction_ <0.01, Table 4). The contrasting results by waist circumference and sex were most pronounced for proximal colon cancer (women with a waist ≥80 cm: adjusted HR [95% CI], 6.56 [5.01–8.59], *P*_interaction_ <0.01; men with a waist <94 cm: adjusted HR [95% CI], 0.13 [0.08–0.22], *P*_interaction_ <0.01). Figure 1 illustrates the multivariable‐adjusted IRRs for the association of LCN2 (per doubling in concentration) with CRC and its subsites after additional adjustment for selected biomarkers. Overall, the observed associations were only slightly altered after including all biomarkers in the model. In sensitivity analyses, excluding participants with a follow‐up time less than 2 years, extreme LCN2 concentrations, or with missing data on covariates, restricting the analysis to postmenopausal women, or using predefined cut‐points, did not substantially change the main findings (Tables S4–S9).

**TABLE 3:** Incidence rate ratios (IRRs)^a^ and 95% confidence intervals (CIs) for the association of LCN2 with colorectal cancer and subsites, overall and by sex.

Outcome	LCN2 _log2_					
	Both sexes		Men		Women	
	IRR (95% CI)	*p‐*value	IRR (95% CI)	*p*‐value	IRR (95% CI)	*p*‐value^b^
Colorectal cancer, *N* (cases/controls)	1267/1267		610/610		657/657	
Model1^c^	1.18 (0.99–1.39)	.06	1.06 (0.86–1.29)	.60	1.39 (1.07–1.80)	.01
Model2^d^	1.16 (0.98–1.37)	.09	1.02 (0.83–1.25)	.87	1.31 (1.00–1.72)	.05
Colon cancer, *N* (cases/controls)	791/791		354/354		437/437	
Model1^c^	1.24 (0.99–1.54)	.06	0.93 (0.68–1.29)	.68	1.58 (1.16–2.14)	.00
Model2^d^	1.26 (1.00–1.59)	.05	0.85 (0.58–1.22)	.38	1.66 (1.20–2.30)	.00
Proximal colon cancer, *N* (cases/controls)	341/341		146/146		195/195	
Model1^c^	1.36 (0.98–1.88)	.07	0.98 (0.61–1.57)	.93	1.82 (1.15–2.89)	.01
Model2^d^	1.40 (0.98–2.02)	.07	0.91 (0.49–1.71)	.77	1.96 (1.15–3.34)	.01
Distal colon cancer, *N* (cases/controls)	393/393		177/177		216/216	
Model1^c^	1.09 (0.79–1.51)	.59	0.88 (0.54–1.42)	.59	1.32 (0.85–2.05)	0.22
Model2^d^	1.10 (0.78–1.55)	.59	0.72 (0.40–1.28)	.26	1.42 (0.88–2.30)	.15
Rectal cancer, *N* (cases/controls)	461/461		246/246		215/215	
Model1^c^	1.11 (0.87–1.41)	.42	1.15 (0.85–1.55)	.36	0.98 (0.60–1.62)	.95
Model2^d^	1.08 (0.85–1.38)	.54	1.16 (0.86–1.56)	.33	0.77 (0.44–1.34)	.36

^a^ Conditional logistic regression was employed to evaluate the association of LCN2 with colorectal cancer risk per doubling of LCN2 concentration.

^b^ Lunn–McNeil competing risk models for CRC showed LCN2 associations with the subsites of proximal colon cancer (*p* <.001), distal colon cancer (*p* <.001), and rectal cancer (*p* <.001) using the chi‐square test.

^c^ Model 1 account for the matching factors: age, sex, study center, follow‐up time since blood collection, time of the day at blood collection and fasting status. Women were further matched by menopausal status, phase of menstrual cycle at blood collection, and postmenopausal women were matched by hormone replacement therapy use.

^d^ Model 2 is based on Model 1, further adjusted for smoking status, alcohol consumption, physical activity, fiber intake, consumption of fruits and vegetables, red and processed meat, fish and shellfish, and waist circumference.

**TABLE 4:** Multivariable‐adjusted hazard ratios (HRs)^a^ and 95% confidence intervals (95% CIs) for the association of LCN2 with colorectal cancer and its subsites, stratified by waist circumference categories.

Outcome	LCN2 _log2_				
	Waist circumference				
	<94 cm (men), <80 cm (women)		≥94 cm (men), ≥80 cm (women)		*p*_nteraction_ ^b^
	HR (95% CI)	*p*‐value	HR (95% CI)	*p‐*value	
Colorectal cancer, *N* (cases/controls)					
Both sexes	516/600		751/667		
	0.80 (0.72–0.88)	<.01	1.41 (1.3–1.53)	<.01	<0.01
Men	239/293		371/317		
	0.48 (0.41–0.57)	<.01	1.22 (1.10–1.36)	<.01	<0.01
Women	277/307		380/350		
	0.92 (0.81–1.05)	.22	2.08 (1.81–2.40)	<.01	<0.01
Colon cancer, *N* (cases/controls)					
Both sexes	319/378		472/413		
	0.86 (0.76–0.98)	.03	1.69 (1.52–1.88)	<.01	0.01
Men	138/176		216/178		
	0.32 (0.25–0.41)	<.01	1.52 (1.32–1.76)	<.01	<0.01
Women	181/202		256/235		
	1.14 (0.98–1.34)	.10	3.18 (2.66–3.79)	<.01	<0.01
Proximal colon cancer, *N* (cases/controls)					
Both sexes	139/158		202/183		
	0.88 (0.72–1.08)	.24	3.19 (2.70–3.77)	<.01	<0.01
Men	56/72		90/74		
	0.13 (0.08–0.22)	<.01	0.75 (0.58–0.98)	.03	<0.01
Women	90/74		112/109		
	1.05 (0.79–1.41)	.72	6.56 (5.01–8.59)	<.01	<0.01
Distal colon cancer, *N* (cases/controls)					
Both sexes	154/201		239/192		
	0.77 (0.61–0.96)	.02	2.24 (1.88–2.67)	<.01	<0.01
Men	66/92		111/85		
	0.18 (0.10–0.30)	<.01	2.14 (1.62–2.84)	<.01	<0.01
Women	88/109		128/107		
	1.58 (1.14–2.18)	.01	5.30 (3.88–7.24)	<.01	<0.01
Rectal cancer, *N* (cases/controls)					
Both sexes	193/219		268/242		
	0.92 (0.77–1.11)	.38	1.20 (1.04–1.38)	.01	0.02
Men	99/115		147/131		
	0.86 (0.65–1.12)	.26	1.1 (0.98–1.42)	.07	0.09
Women	94/104		121/111		
	0.89 (0.67–1.17)	.40	1.01 (0.78–1.31)	.92	0.05

^a^ Based on weighted Cox proportional hazard regression using inverse probability weighting to approximate the full cohort to evaluate the association of LCN2 with colorectal cancer risk per doubling of LCN2 concentration. HRs adjusted for age, sex, study center, follow‐up time since blood collection, time of the day at blood collection and fasting status. Women were further matched by menopausal status, phase of menstrual cycle at blood collection, and postmenopausal women were matched by hormone replacement therapy use and is further adjusted for smoking status, alcohol consumption, physical activity, fiber intake, consumption of fruits and vegetables, red and processed meat, fish and shellfish, and waist circumference.

^b^ *P* values for interaction were calculated with the Wald‐test for a multiplicative interaction term of LCN2 and the continuous waist circumference variable.

## 4 DISCUSSION

In this prospective cohort study, pre‐diagnostic LCN2 concentrations were positively associated with colon cancer, particularly occurring in the proximal colon. Furthermore, these associations were more pronounced in women and among individuals with abdominal adiposity. Conversely, male individuals with a low waist circumference presented themselves with an inverse association with colorectal cancer, most pronounced in proximal colon cancer.

To our knowledge, this is the first prospective analysis of the association between pre‐diagnostic LCN2 concentrations and risk of incident CRC. Despite the role of circulating LCN2 as a diagnostic biomarker for CRC has been proposed by case–control studies,^14^ its implication in the development of CRC in a large prospective study setting has not been explored. Our analyses revealed a positive association between the LCN2 concentration and CRC that was especially pronounced in women and for (proximal) colon cancer. It has long been recognized that cancers arising in different anatomical sites of the colorectum represent etiologically and clinically different subtypes characterized by different sex‐specific incidence rates, risk factor profiles, and distinct molecular and clinical characteristics.^32^ There are notable distinctions between proximal and distal CRCs, such that proximal carcinomas are more commonly reported in women and older individuals.^33^, ^34^ In recent years there has been a rise in the incidence rates of proximal colon cancers.^35^ Proximal tumors are also more likely to exhibit hypermethylated DNA and to have elevated mutation rates characterized by microsatellite instability, CpG island methylator phenotype, and *BRAF* mutation predisposing formation of sessile serrated polyps.^36^, ^37^ In contrast, distal colon cancers more commonly occur in men and are characterized by chromosomal instability.^36^ Proximal and distal colon sites are further characterized by different mucosal microbial community environments with the proximal colon having higher immune activity compared to distal colon.^38^ Pathogen‐induced infection may trigger the LCN2 signaling pathway, including an increase in LCN2 expression, subsequently leading to apoptosis, oxidative stress, and site‐specific inflammation promoting tumorigenesis.^39^ Other pathways in which LCN2 has been implicated, including regulation of iron homeostasis,^40^ retinol metabolism^41^ and epithelial–mesenchymal transition,^42^ may also provide explanations for the observed associations. LCN2 triggered by bacterial infection can bind to bacterial iron‐loaded siderophores and enhance tumor cell proliferation.^43^ LCN2 was also shown to play a role in an iron‐dependent mode of cell death called ferroptosis characterized by disordered iron metabolism and oxidative stress.^13^, ^44^ During initial stages of carcinogenesis, pro‐inflammatory cytokines promote LCN2 production and iron sequestration in macrophages and facilitate accelerated production of reactive oxygen species as a first‐line anti‐tumor defense mechanism.^45^ Iron‐regulatory mechanisms in the tumor microenvironment have been recently suggested to play a significant role in colorectal carcinogenesis, particularly affecting proximal colon.^46^, ^47^ It should be noted though that clinical studies provided inconsistent results regarding site‐specific expression of LCN2 in tumor tissues, with some studies reporting no difference by cancers site,^12^ while others showed an overexpression of LCN2 in proximal as compared to distal and rectal tumor tissues.^48^ Our study was based on pre‐diagnostic concentrations of LCN2 and focused on cancer development, whereas its role in cancer progression and its utility as prognostic biomarker requires future evaluation. Future studies are warranted to replicate these findings explore the potential of LCN2 as an early risk biomarker for colon cancer and elucidate the mechanisms behind the observed sex‐ and site‐specific associations.

Our study further revealed an association between elevated LCN2 and risk of colon cancer in participants characterized by abdominal adiposity. In contrast, an inverse association was seen in individuals without abdominal adiposity that was particularly strong in men. Previous studies including EPIC data, showed that abdominal adiposity was associated with the occurrence of CRC independent of general obesity as reflected by a high body mass index.^49^ Abdominal adiposity may reflect metabolic health status more precisely compared to general obesity.^50^ Visceral adipose tissue is metabolically more active compared to subcutaneous fat, secreting a variety of inflammatory mediators including LCN2.^51^, ^52^, ^53^ Experimental in vitro and animal research has demonstrated that LCN2 is predominantly expressed in the visceral adipose tissue when triggered by inflammatory stimuli, including lipopolysaccharides and interleukin 1 beta.^9^, ^10^ In human studies, LCN2 mRNA is over‐expressed in adipose tissue of obese patients, and higher concentrations of LCN2 were more strongly associated with excessive visceral fat as compared to total body fat.^54^, ^55^ A strong positive correlation was also found between LCN2 expression and the mean diameter of adipocytes in visceral adipose tissue.^56^ Animal studies further uncovered the possible roles of LCN2 in systemic insulin sensitivity and glucose homeostasis.^9^, ^10^ Compared to animal research, the role of circulating LCN2 in human studies has been less explored. Most studies have suggested that LCN2 concentrations have been associated with metabolic biomarkers, such as fasting glucose, homeostasis model assessment of insulin resistance index, and hs(CRP) after controlling for body mass index, suggesting that it may play an independent role in the regulation of insulin resistance and inflammation.^10^, ^57^ Obese and type 2 diabetic patients have been characterized with increased levels of LCN2 in both circulation and adipose tissue and elevated serum lipocalin‐2 was independently associated with impaired glucose regulation and type 2 diabetes.^58^ Moreover, LCN2 was shown to suppress insulin‐sensitizing molecules, such as peroxisome proliferator‐activated receptor gamma and adiponectin gene expression.^10^ Treatment with the PPARγ agonist rosiglitazone was effective in reducing LCN2 levels in diabetic patients, suggesting its possible role in the regulation of human adipogenesis.^59^ Synthetic glucocorticoids dose‐dependently increased LCN2 gene and protein expression in adipose tissue from female donors but had no effect in adipose tissue obtained from males. In females, LCN2 gene expression correlated positively with markers of obesity, insulin resistance, and hyperglycemia.^59^ Previous research has further suggested a possible role of sex steroid hormones, such as estrogen, in the regulation of LCN2 in adipose tissue.^60^ Estrogen signaling plays a critical role in the maintenance of metabolic conditions such as obesity and insulin resistance. In postmenopausal women, reduced estrogen synthesis has been linked to impaired glucose and lipid metabolism, distribution of fat from peripheral to central depots, and insulin resistance.^61^, ^62^ To explore possible influence of menopausal status on the associations between LCN2 and colon cancer, we have conducted a sensitivity analysis examining data from postmenopausal women only. However, results were not changed suggesting that LCN2 is associated with colon cancer independent of menopausal status. Our findings pointing to a possible protective role of LCN2 in lean individuals, and in men in particular, deserve to be further replicated in future research. Circulating LCN2 was shown to promote the browning of fat tissue^63^ and to regulate glucose intolerance and food intake in mice,^63^ as well as serve as an anorexigenic signal in obese monkeys.^64^ Further studies combining lines of research from animal models and humans would be warranted to characterize the pathophysiological properties of LCN2 and explore its potential mediating role on the association between adiposity and colon cancer.

Our study has several strengths. We included a large number of participants from several European countries in a prospective population‐based cohort study, allowing for detailed analyses by sex and cancer subsites. To account for the reduced power in the analyses stratified by waist circumference, we have employed inverse probability weighting for weighted Cox regression to improve statistical efficiency.

Our study also has several limitations. First, data on several covariates in adjustment models, including anthropometry, lifestyle, and other biomarkers were partly incomplete. Nevertheless, we used a sophisticated imputation method based on a random forest algorithm, suitable for mixed and complex data, to properly account for missing information. We also conducted analyses based on a sample of participants with complete data and the results remained unchanged. Second, the blood samples used for the measurement of LCN2 have been collected and stored over longer time periods before measurement. The analysis was also based on single measurements of LCN2. However, previous methodological studies did not suggest storage time to influence LCN2 concentrations and our previous assessment showed that LCN2 measurements with the same assay are relatively stable over time.^21^ Third, the study was based on incident cases of CRC and matched controls. It cannot be ruled out that some of the cases had prevalent but undiagnosed CRC at recruitment. However, sensitivity analyses excluding the first 2 years of study follow‐up did not produce substantially different results arguing against potential influence of reverse causality in the analyses.

In summary, in this prospective cohort study, higher pre‐diagnostic concentrations of LCN2 were associated with higher risk of colon cancer, particularly in the proximal colon. The elevated colon cancer risk was especially pronounced in women and among individuals with higher degree of abdominal adiposity. Further studies are warranted to confirm these results and to shed light on the pathophysiological pathways explaining the observed sex‐ and site‐specific associations.

## AUTHOR CONTRIBUTIONS

**Robin Reichmann:** Formal analysis; writing – original draft; writing – review and editing. **Katharina Nimptsch:** Writing – review and editing. **Tobias Pischon:** Writing – review and editing. **Marc J. Gunter:** Writing – review and editing. **Mazda Jenab:** Writing – review and editing. **Anne Kirstine Eriksen:** Writing – review and editing. **Anne Tjønneland:** Writing – review and editing. **Jürgen Janke:** Writing – review and editing. **Verena Katzke:** Writing – review and editing. **Rudolf Kaaks:** Writing – review and editing. **Matthias B. Schulze:** Writing – review and editing. **Fabian Eichelmann:** Writing – review and editing. **Giovanna Masala:** Writing – review and editing. **Sabina Sieri:** Writing – review and editing. **Fabrizio Pasanisi:** Writing – review and editing. **Rosario Tumino:** Writing – review and editing. **Maria Teresa Giraudo:** Writing – review and editing. **Joseph Rothwell:** Writing – review and editing. **Gianluca Serveri:** Writing – review and editing. **Paula Jakszyn:** Writing – review and editing. **Maria‐Jose Sánchez:** Writing – review and editing. **Pilar Amiano:** Writing – review and editing. **Sandra M. Colorado‐Yohar:** Writing – review and editing. **Marcela Guevara:** Writing – review and editing. **Bethany van Guelpen:** Writing – review and editing. **Elom K. Aglago:** Writing – review and editing. **Alicia K. Heath:** Writing – review and editing. **Karl Smith‐Byrne:** Writing – review and editing. **Elisabete Weiderpass:** Writing – review and editing. **Krasimira Aleksandrova:** Conceptualization; formal analysis; investigation; methodology; project administration; supervision; writing – original draft; writing – review and editing.

## FUNDING INFORMATION

The coordination of EPIC is financially supported by International Agency for Research on Cancer (IARC) and by the Department of Epidemiology and Biostatistics, School of Public Health, Imperial College London which has additional infrastructure support provided by the NIHR Imperial Biomedical Research Centre (BRC). The national cohorts are supported by: Danish Cancer Society (Denmark); Ligue Contre le Cancer, Institut Gustave Roussy, Mutuelle Générale de l'Education Nationale, Institut National de la Santé et de la Recherche Médicale (INSERM) (France); German Cancer Aid, German Cancer Research Center (DKFZ), German Institute of Human Nutrition Potsdam‐Rehbruecke (DIfE), Federal Ministry of Education and Research (BMBF) (Germany); Associazione Italiana per la Ricerca sul Cancro‐AIRC‐Italy, Compagnia di SanPaolo and National Research Council (Italy); Dutch Ministry of Public Health, Welfare and Sports (VWS), Netherlands Cancer Registry (NKR), LK Research Funds, Dutch Prevention Funds, Dutch ZON (Zorg Onderzoek Nederland), World Cancer Research Fund (WCRF), Statistics Netherlands (The Netherlands); Health Research Fund (FIS)—Instituto de Salud Carlos III (ISCIII), Regional Governments of Andalucía, Asturias, Basque Country, Murcia and Navarra, and the Catalan Institute of Oncology—ICO (Spain); Swedish Cancer Society, Swedish Research Council, Region Skåne and Region Västerbotten (Sweden); Cancer Research UK (14,136 to EPIC‐Norfolk; C8221/A29017 to EPIC‐Oxford), Medical Research Council (1,000,143 to EPIC‐Norfolk; MR/M012190/1 to EPIC‐Oxford) (United Kingdom).

## CONFLICT OF INTEREST STATEMENT

The authors declare no potential conflict of interest.

## ETHICS STATEMENT

Ethical review boards from IARC and local participating centers approved the study. Written informed consent was obtained from all individual participants included in the study.

## Supporting information


**Data S1.** Supporting Information.

## Data Availability

This study is based on EPIC data. The EPIC data are available for external investigators who seek to answer important questions on health and disease in the context of research projects that are consistent with the legal and ethical standard practices of IARC/WHO and the EPIC Centres, and the analyses were conducted by (or in collaboration with) one or more EPIC investigators. All source code is publicly available on Github: https://github.com/EpiUser/Lipocalin-2-CRC.git.
