# Supplementary material for: Sex‐ and site‐specific associations of circulating lipocalin 2 and incident colorectal cancer: Results from the EPIC cohort
Source: Int J Cancer. 2024 Nov 7;156(5):930–42. doi: 10.1002/ijc.35205 (PMC11701386; doi:10.1002/ijc.35205)
Supplement: Supplementary file 1 — Data S1. Supporting Information. [file IJC-156-930-s001.pdf]

Sex- and site-specific associations of circulating lipocalin 2 and incident colorectal cancer: Results from the EPIC cohort

Robin Reichman, Katharina Nimptsch, Tobias Pischon, Marc J. Gunter, Mazda Jenab, Anne Kirstine Eriksen, Anne Tjønneland, Jürgen Janke, Verena Katzke, Rudolf Kaaks, Matthias B. Schulze, Fabian Eichelmann, Giovanna Masala, Vittorio Krogh, Fabrizio Pasanisi, Rosario Tumino, Maria Teresa Giraudo, Joseph Rothwell, Gianluca Serveri, Paula Jakszyn, Maria-Jose Sánchez, Pilar Amiano, Sandra M. Colorado-Yohar, Marcela Guevara, Bethany van Guelpen, Elom K. Aglago, Alicia K. Heath, Karl Smith-Byrne, Elisabete Weiderpass, Krasimira Aleksandrova

Supplementary Information

Table of Contents:

Figure S1. Flowchart of the nested case-control study in EPIC.

Figure S2a. Distribution of LCN2 concentration in control participants (n=1,267).

Figure S2b. Distribution of LCN2 concentration in all participants, stratified by sex and cancer status.

Figure S3. Incidence rate ratios (IRRs) and 95% confidence intervals for the association between LCN2 concentrations and colorectal cancer and subsites modelled with restricted cubic splines with 3 knots, overall and by sex.

Table S1. Missing data by case and control status.

Table S2. Spearman correlation of LCN2 and continuous covariates, partially adjusted for sex and age at blood collection. Confidence intervals were calculated with Fisher transformation.

Table S3. Incidence rate ratios (IRRs) and 95% confidence intervals (95% CIs) of LCN2 quartile categories with colorectal cancer and subsites, overall, and by sex.

Table S4. Hazard Ratios (HR) and 95% confidence intervals (95% CIs) of LCN2 and CRC and subsites, excluding cases with a follow-up of <2 years (N_{cases}= 231) and matched controls ($N_{controls}$ = 231), stratified by sex and waist circumference.

Table S5. Hazard Ratios (HR) and 95% confidence intervals (95% CIs) of LCN2 and CRC and subsites, excluding cases (N_{cases} = 53) and matched controls ($N_{controls}$ = 53) with an LCN2 concentration below the 1st and above the 99th sex-specific LCN2 concentration-percentile, stratified by sex and waist circumference.

Table S6. Hazard Ratios (HRs) and 95% confidence intervals (95% CIs) of LCN2 and CRC and subsites, excluding cases (N_{cases} = 77) and matched controls ($N_{controls}$ = 77) with missing covariates, stratified by sex and waist circumference.

Table S7. Hazard Ratios (HRs) and 95% confidence intervals (95% CIs) of LCN2 and CRC and subsites, only including postmenopausal women cases (N_{cases} = 509) and matched controls ($N_{controls}$ = 509), stratified by waist circumference.

Table S8. Multivariable-adjusted hazard ratios a (HRs) and 95% confidence intervals (95% CIs) for the association of LCN2 with colorectal cancer comparing high vs. low LCN2 levels based on a-priori defined cut-points.

Table S9. Multivariable-adjusted hazard ratios a (HRs) and 95% confidence intervals (95% CIs) for the association of LCN2 with colorectal cancer comparing high vs. low LCN2 levels according to predefined cu-points, stratified by waist circumference categories.

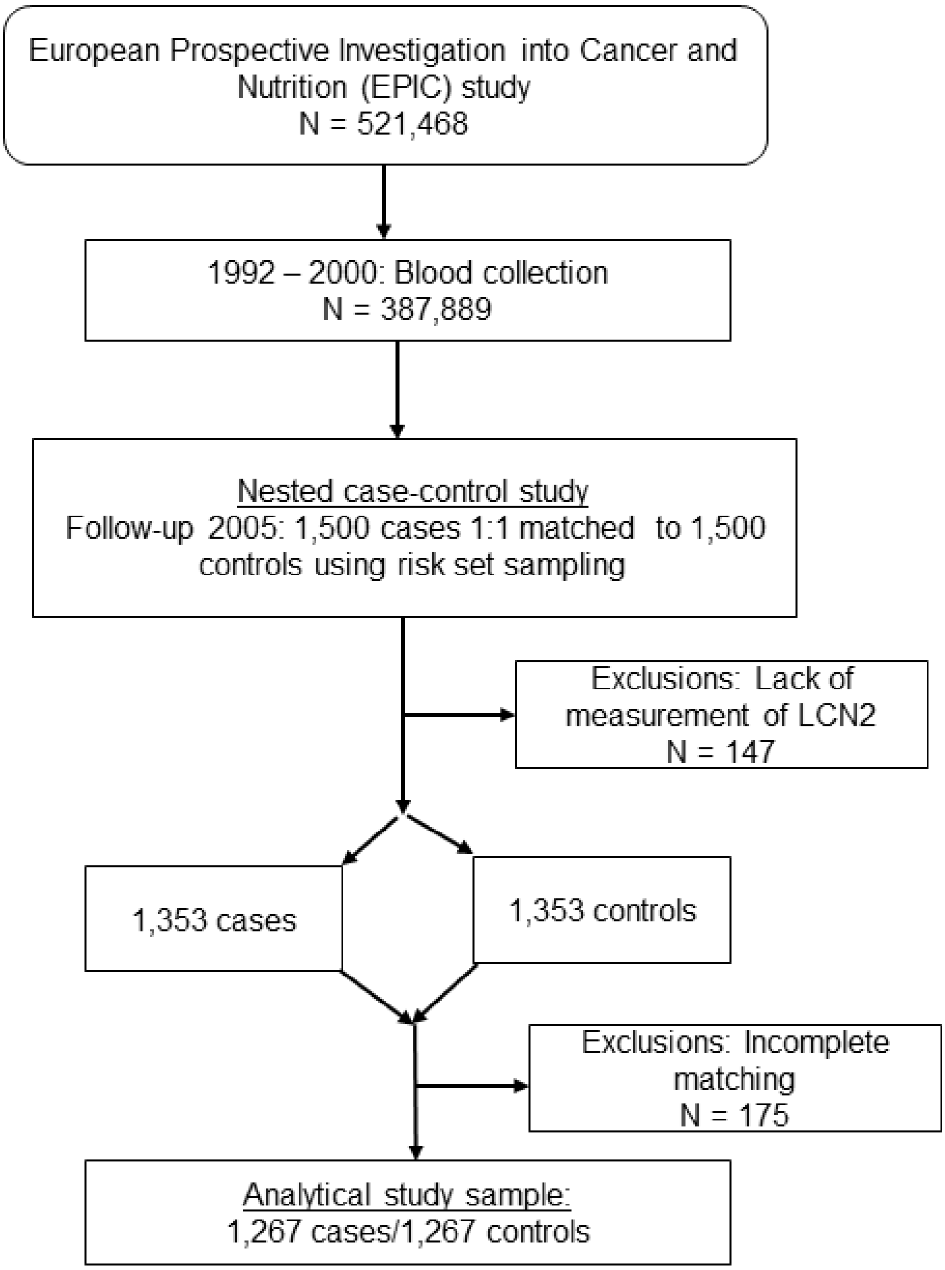

Figure S1. Flowchart of the nested case-control study in EPIC.

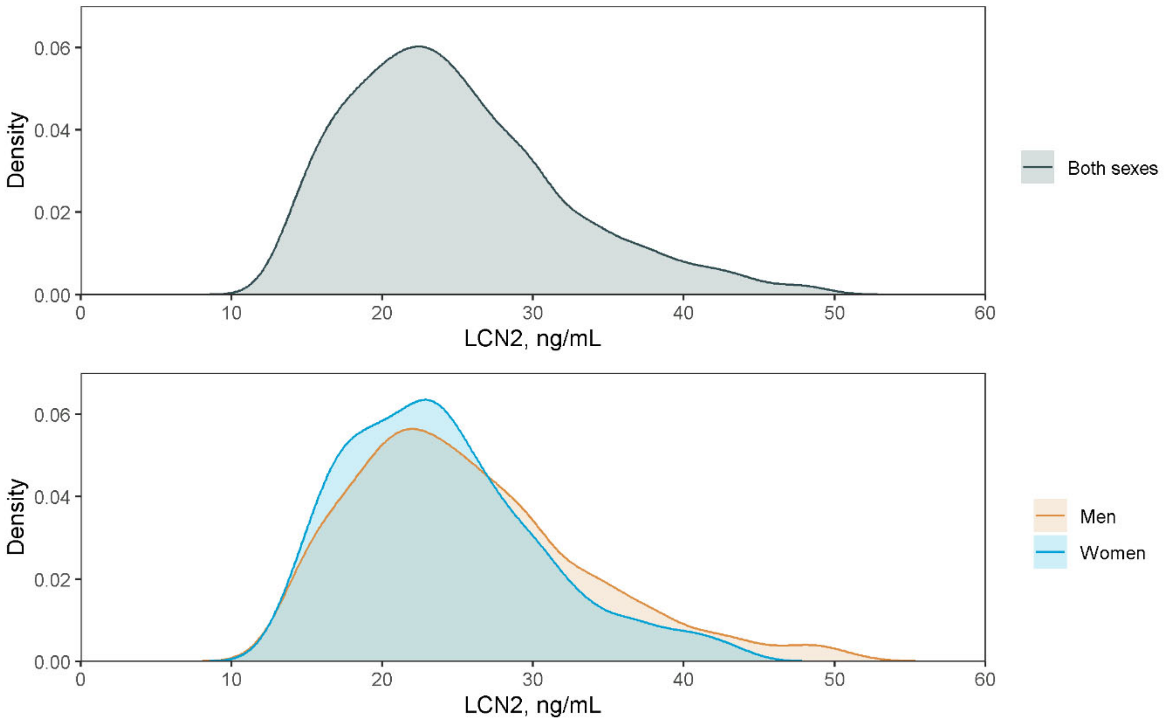

Figure S2a. Distribution of LCN2 concentration in control participants (n=1,267). $p_{\text{difference by sex}}$ based on Wilcoxon's signed rank test: <0.001.

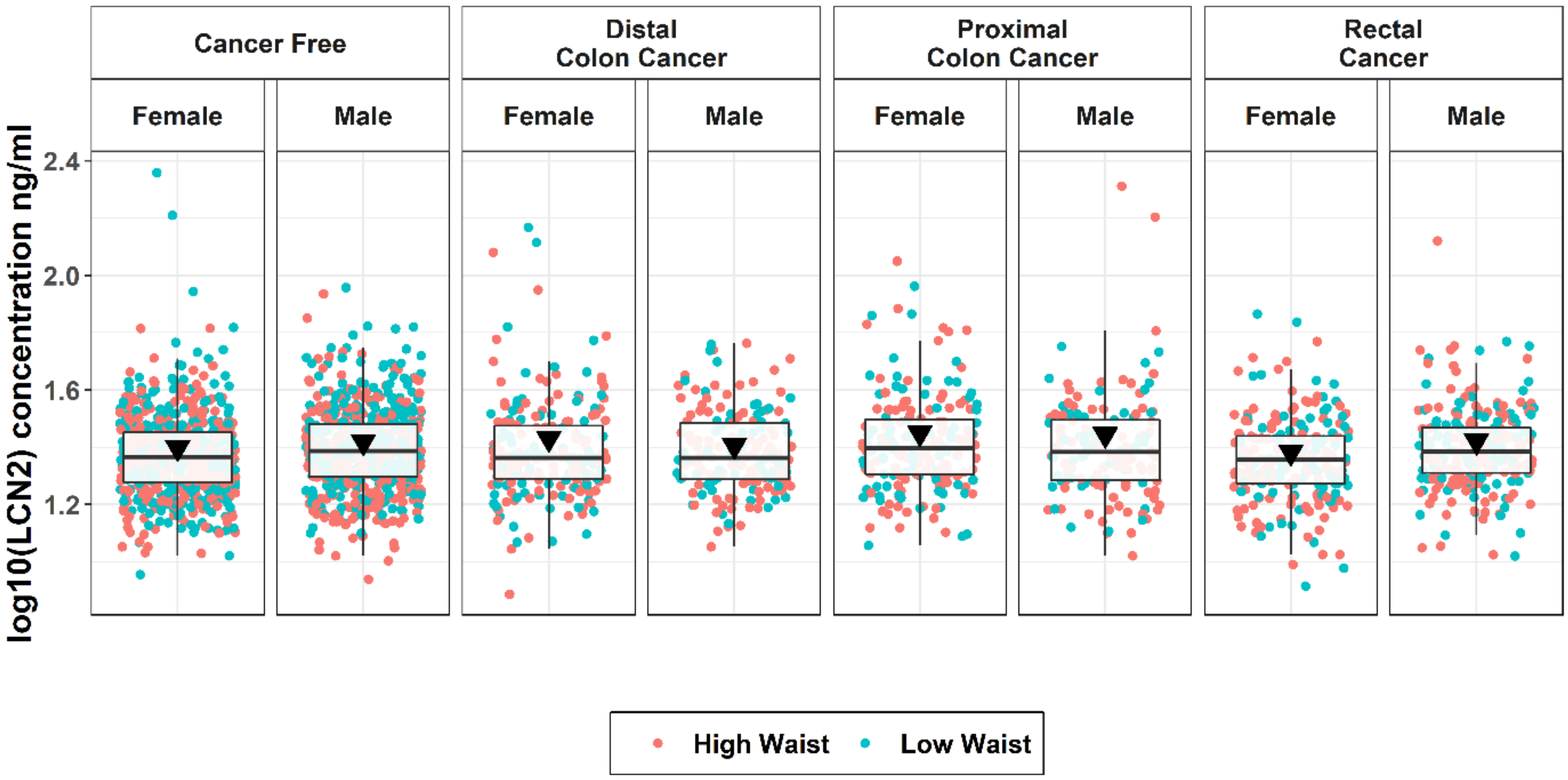

Figure S2b. Distribution of LCN2 concentration in all participants, stratified by sex and cancer status. The triangles mark each respective mean value.

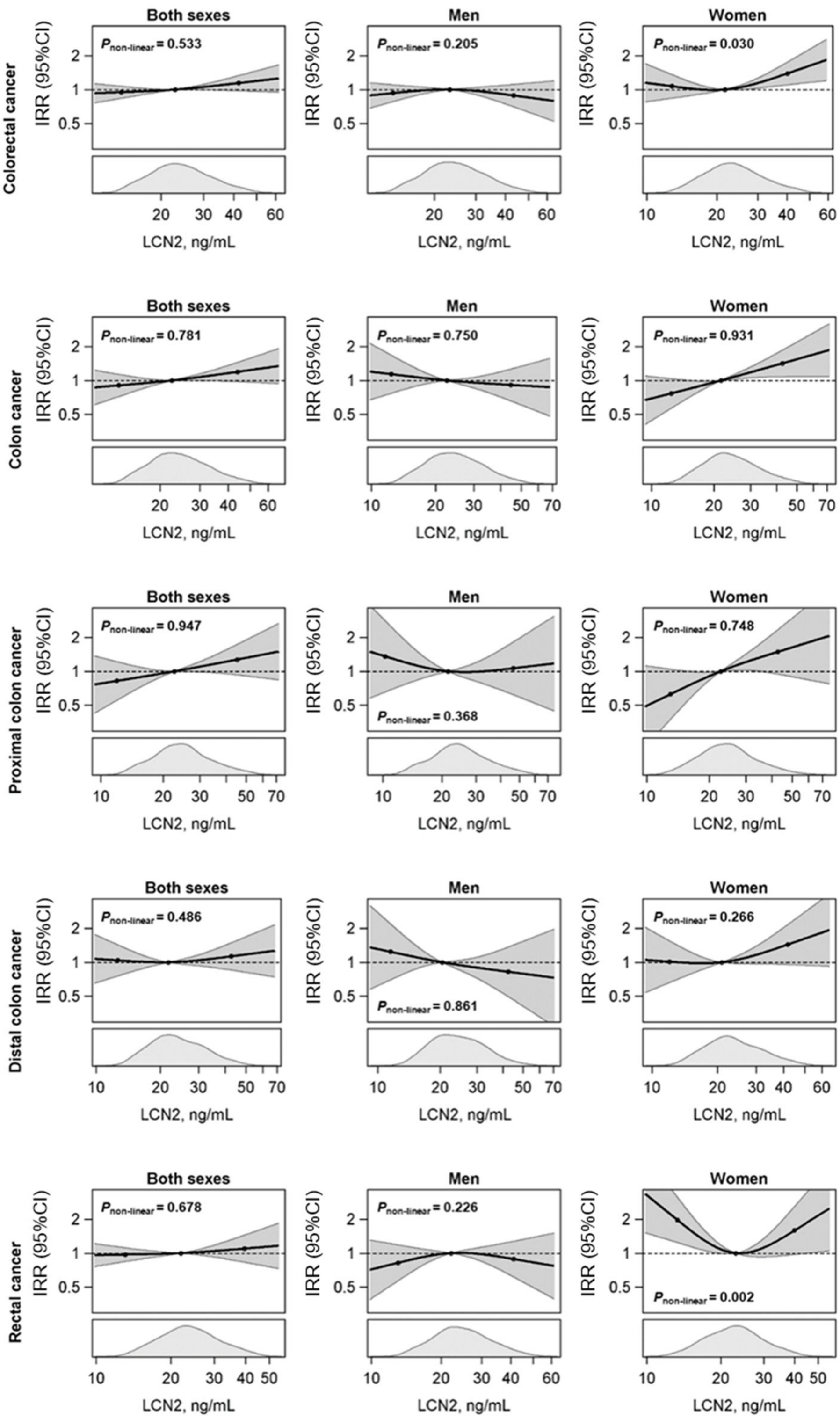

Figure S3. Incidence rate ratios (IRRs) and 95% confidence intervals for the association between LCN2 concentrations and colorectal cancer and subsites modelled with restricted cubic splines with 3 knots, overall and by sex.

Incidence rate ratios (IRRs) accounting for matching factors: age, sex, study center, follow-up time since blood collection, time of the day at blood collection and fasting status. Women were further matched by menopausal status, phase of menstrual cycle at blood collection, postmenopausal women were matched by hormone replacement therapy use. The model was further adjusted for smoking status, alcohol consumption, physical activity, fiber intake, consumption of fruits and vegetables, red and processed meat, fish and shellfish, and waist circumference.

Table S1. Missing data by case and control status.

Characteristics	Cases (N = 1,267)		Controls (N = 1,267)	
	N	**%**	***N***	**%**
BMI	6	0.47	7	0.55
Waist circumference	70	5.52	67	5.29
Education	11	0.87	9	0.71
Physical activity	74	5.84	79	6.24
Diet	4	0.32	0	0.00
CRP	407	32.12	546	43.09
Non-HMW adiponectin	491	38.75	623	49.17
HDL cholesterol	408	32.20	547	43.17
ROM	402	31.73	550	43.41
Neopterin	395	31.18	699	55.17

Abbreviations: BMI, body mass index; CRP, C-reactive protein; HDL, high-density lipoprotein; HMW, high molecular weight; ROM, reactive oxygen metabolites.

Table S2. Spearman correlation of LCN2 and continuous covariates, partially adjusted for sex and age at blood collection. Confidence intervals were calculated with Fisher transformation.

Characteristics	Spearman ρ (95% confidence interval)
C-reactive protein	0.18 (0.12 – 0.23)
Non-HMW adiponectin	-0.10 (-0.16 – -0.05)
TNF alpha	0.26 (0.21 – 0.31)
HDL cholesterol	-0.16 (-0.21 – -0.11)
ROM	0.15 (0.09 – 0.20)
Neopterin	0.14 (0.09 – 0.19)

Abbreviations: HDL, high-density lipoprotein; HMW, high molecular weight; LCN2, lipocalin 2; ROM, reactive oxygen metabolites; TNF, tumor necrosis factor.

Table S3. Incidence rate ratios (IRRs)[a] and 95% confidence intervals (95% CIs) of LCN2 quartile categories with colorectal cancer and subsites, overall, and by sex.

LCN2, ng/mL	LCN2 quartile categories (median (range))				P_{trend} [b]
	**1**<br>**16.57 (7.7-19.2 ng/ml)**	**2**<br>**21.50 (19.2 – <23.6 ng/ml)**	**3**<br>**26.00 (23.6 – <29.0 ng/ml)**	**4**<br>**34.41 (29.0-228.3 ng/ml)**	
**Colorectal cancer**					
**Both sexes, *N* (cases/controls)**	287/316	350/315	288/315	342/321	
IRR (95%CI)	1 (Ref.)	1.23 (0.97 – 1.56)	0.98 (0.77 – 1.25)	1.13 (0.88 – 1.45)	0.64
**Men, *N* (cases/controls)**	135/138	163/144	145/151	167/177	
IRR (95%CI)	1 (Ref.)	1.18 (0.82 – 1.69)	0.87 (0.60 – 1.26)	0.84 (0.58 – 1.20)	0.16
**Women, *N* (cases/controls)**	152/178	187/171	143/164	175/144	
IRR (95%CI)	1 (Ref.)	1.24 (0.89 – 1.72)	0.98 (0.70 – 1.37)	1.44 (0.99 – 2.09)	0.13
**Colon cancer**					
**Both sexes, *N* (cases/controls)**	179/196	215/199	171/191	226/205	
IRR (95%CI)	1 (Ref.)	1.25 (0.93 – 1.67)	1.01 (0.74 – 1.38)	1.24 (0.90 – 1.70)	0.33
**Men, *N* (cases/controls)**	88/78	90/87	79/89	97/100	
IRR (95%CI)	1 (Ref.)	1.03 (0.64 – 1.64)	0.69 (0.42 – 1.14)	0.72 (0.44 – 1.19)	0.19
**Women, *N* (cases/controls)**	91/118	125/102	92/102	129/105	
IRR (95%CI)	1 (Ref.)	1.54 (1.04 – 2.29)	1.30 (0.84 – 2.00)	1.85 (1.18 – 2.91)	0.02
**Proximal colon cancer**					
**Both sexes, *N* (cases/controls)**	75/75	82/89	86/92	98/85	
IRR (95%CI)	1 (Ref.)	0.94 (0.58 – 1.53)	1.01 (0.62 – 1.63)	1.19 (0.70 – 2.02)	0.45
**Men, *N* (cases/controls)**	38/28	33/39	35/38	40/41	
IRR (95%CI)	1 (Ref.)	0.43 (0.18 – 1.02)	0.49 (0.20 – 1.21)	0.43 (0.16 – 1.13)	0.17
**Women, *N* (cases/controls)**	37/47	49/50	51/54	58/44	
IRR (95%CI)	1 (Ref.)	1.43 (0.74 – 2.77)	1.50 (0.78 – 2.88)	2.14 (1.01 – 4.54)	0.06
**Distal colon cancer**					

**Both sexes, *N* (cases/controls)**	93/103	124/98	68/88	108/104	
IRR (95%CI)	1 (Ref.)	1.51 (1.00 – 2.28)	0.79 (0.49 – 1.27)	1.10 (0.70 – 1.73)	0.92
**Men, *N* (cases/controls)**	43/42	52/42	35/44	47/49	
IRR (95%CI)	1 (Ref.)	1.54 (0.75 – 3.13)	0.51 (0.24 – 1.11)	0.74 (0.36 – 1.54)	0.23
**Women, *N* (cases/controls)**	50/61	72/56	33/44	61/55	
IRR (95%CI)	1 (Ref.)	1.55 (0.88 – 2.74)	1.04 (0.52 – 2.08)	1.43 (0.76 – 2.69)	0.46
**Rectal cancer**					
**Both sexes, *N* (cases/controls)**	109/115	136/119	106/125	110/102	0.76
IRR (95%CI)	1 (Ref.)	1.15 (0.76 – 1.73)	0.81 (0.53 – 1.22)	1.03 (0.66 – 1.62)	
**Men, *N* (cases/controls)**	48/57	71/60	61/64	66/65	
IRR (95%CI)	1 (Ref.)	1.46 (0.81 – 2.61)	1.09 (0.59 – 2.02)	1.08 (0.59 – 1.97)	0.81
**Women, *N* (cases/controls)**	61/58	65/59	45/61	44/37	
IRR (95%CI)	1 (Ref.)	0.94 (0.49 – 1.79)	0.53 (0.28 – 0.98)	1.01 (0.45 – 2.23)	0.45

[a] Multivariable-adjusted IRRs considering matching factors: age, sex, study center, follow-up time since blood collection, time of the day at blood collection and fasting status. Women were further matched by menopausal status, phase of menstrual cycle at blood collection; postmenopausal women were matched by hormone replacement therapy use. The model was further adjusted for smoking status, alcohol consumption, physical activity, fiber intake, consumption of fruits and vegetables, red and processed meat, fish and shellfish, and waist circumference.

[b] *P* values for trend were calculated with the Wald test on a multivariable-adjusted model with the sex-specific quartile categories modelled linearly as the sex-specific median of each category.

Table S4. Hazard Ratios (HR)[a] and 95% confidence intervals (95% CIs) of LCN2 and CRC and subsites, excluding cases with a follow-up of <2 years (N_{cases}= 231) and matched controls ($N_{controls}$ = 231), stratified by sex and waist circumference.

**Outcome**	**LCN2 log_2**				**$P_{interaction}$**[b]
	**Waist circumference**				
	**<94cm (men), <80cm (women)**		**≥94cm (men), ≥80cm (women)**		
	**HR (95% CI)**	***P***	**HR (95% CI)**	***P***	
**Colorectal cancer, *N* (cases/controls)**					
Both sexes	417/493		619/543		
	0.72 (0.64 – 0.81)	<0.01	1.22 (1.11 – 1.34)	<0.01	<0.01
Men	190/241		309/258		
	0.42 (0.35 – 0.51)	<0.01	0.96 (0.85 – 1.09)	0.56	<0.01
Women	227/252		310/285		
	0.81 (0.70 – 0.95)	<0.01	1.75 (1.49 – 2.06)	<0.01	<0.01
**Colon cancer, *N* (cases/controls)**					
Both sexes	261/316		394/339		
	0.80 (0.69 – 0.93)	<0.01	1.58 (1.40 – 1.78)	<0.01	0.03
Men	111/150		184/145		
	0.23 (0.17 – 0.30)	<0.01	1.237 (1.17 – 1.60)	<0.01	<0.01
Women	150/166		210/194		
	1.23 (1.01 – 1.50)	0.04	2.36 (1.93 – 2.89)	<0.01	<0.01
**Proximal colon cancer, *N* (cases/controls)**					
Both sexes	109/130		174/153		
	0.56 (0.44 – 0.72)	<0.01	2.96 (2.45 – 3.57)	<0.01	<0.01
Men	42/59		79/62		
	0.02 (0.01 – 0.04)	<0.01	0.92 (0.69 – 1.23)	0.58	<0.01
Women	67/71		95/91		

	0.88 (0.60 – 1.31)	0.55	5.94 (4.35 – 8.12)	<0.01	<0.01
**Distal colon cancer, *N* (cases/controls)**					
Both sexes	130/173		197/154		
	0.88 (0.69 – 1.13)	0.31	1.94 (1.61 – 2.34)	<0.01	<0.01
Men	56/82		93/67		
	0.12 (0.07 – 0.23)	<0.01	2.43 (1.83 – 3.21)	<0.01	<0.01
Women	74/91		104/87		
	2.37 (1.61 – 3.48)	<0.01	4.62 (3.06 – 6.96)	<0.01	<0.01
**Rectal cancer, *N* (cases/controls)**					
Both sexes	152/174		271/195		
	0.86 (0.70 – 1.07)	0.18	1.08 (0.91 – 1.29)	0.39	0.23
Men	77/89		120/108		
	1.02 (0.74 – 1.41)	0.91	0.94 (0.73 – 1.20)	0.61	0.31
Women	75/85		97/87		
	0.71 (0.51 – 0.98)	0.04	1.21 (0.90 – 1.62)	0.21	<0.01

[a] Based on weighted Cox proportional hazard regression using inverse probability weighting to approximate the full cohort to evaluate the association of LCN2 with colorectal cancer risk per doubling of LCN2 concentration. HRs were adjusted for age, sex, study center, follow-up time since blood collection, time of the day at blood collection, fasting status, smoking status, alcohol consumption, physical activity, fiber intake, consumption of fruits and vegetables, red and processed meat, fish and shellfish, and waist circumference. Women were additionally matched by menopausal status, and phase of menstrual cycle at blood collection; postmenopausal women by hormone replacement therapy use.

[b] *P* values for interaction were calculated with the Wald-test for a multiplicative interaction term of LCN2 and the continuous waist circumference variable.

Table S5. Hazard Ratios (HR)[a] and 95% confidence intervals (95% CIs) of LCN2 and CRC and subsites, excluding cases (N_{cases} = 53) and matched controls ($N_{controls}$ = 53) with an LCN2 concentration below the 1st and above the 99th sex-specific LCN2 concentration-percentile, stratified by sex and waist circumference.

Outcome	LCN2 log_2				$P_{interaction}$[b]
	Waist circumference				
	<94cm (men), <80cm (women)		≥94cm (men), ≥80cm (women)		
	**HR (95% CI)**	***P***	**HR (95% CI)**	***P***	
**Colorectal cancer, *N* (cases/controls)**					
Both sexes	495/576		719/638		
	0.74 (0.65 – 0.83)	<0.01	1.46 (1.32 – 1.60)	<0.01	<0.01
Men	232/284		352/300		
	0.46 (0.38 – 0.55)	<0.01	1.29 (1.13 – 1.48)	<0.01	<0.01
Women	263/292		367/338		
	1.00 (0.84 – 1.20)	0.97	1.85 (1.60 – 2.14)	<0.01	<0.01
**Colon cancer, *N* (cases/controls)**					
Both sexes	309/360		450/399		
	0.92 (0.79 – 1.07)	0.28	1.61 (1.43 – 1.81)	<0.01	0.33
Men	136/170		204/170		
	0.35 (0.27 – 0.47)	<0.01	1.47 (1.23 – 1.75)	<0.01	<0.01
Women	173/190		246/229		
	1.48 (1.20 – 1.82)	<0.01	2.56 (2.13 – 3.08)	<0.01	<0.01
**Proximal colon cancer, *N* (cases/controls)**					
Both sexes	132/153		194/123		
	1.05 (0.83 – 1.33)	0.68	2.50 (2.06 – 3.04)	<0.01	<0.01
Men	54/71		85/68		
	0.10 (0.06 – 0.18)	<0.01	0.55 (0.39 – 0.76)	<0.01	<0.01
Women	78/82		109/105		

	2.95 (1.92 – 4.53)	<0.01	4.80 (3.61 – 6.39)	<0.01	<0.01
**Distal colon cancer, *N* (cases/controls)**					
Both sexes	152/189		227/190		
	0.76 (0.59 – 0.99)	0.04	2.60 (2.13 – 3.17)	<0.01	<0.01
Men	66/87		104/83		
	0.23 (0.12 – 0.44)	<0.01	2.87 (2.14 – 3.85)	<0.01	0.02
Women	86/102		123/107		
	2.85 (1.90 – 4.29)	<0.01	4.68 (3.25 – 6.76)	<0.01	<0.01
**Rectal cancer, *N* (cases/controls)**					
Both sexes	183/214		258/227		
	0.77 (0.63 – 0.96)	0.02	1.37 (1.16 – 1.62)	<0.001	0.093
Men	95/113		140/122		
	0.79 (0.58 – 1.07)	0.13	1.43 (1.11 – 1.85)	0.006	0.869
Women	88/101		118/105		
	0.59 (0.42 – 0.82)	<0.01	1.09 (0.83 – 1.43)	0.535	0.006

[a] Based on weighted Cox proportional hazard regression using inverse probability weighting to approximate the full cohort to evaluate the association of LCN2 with colorectal cancer risk per doubling of LCN2 concentration. HRs were adjusted for age, sex, study center, follow-up time since blood collection, time of the day at blood collection, fasting status, smoking status, alcohol consumption, physical activity, fiber intake, consumption of fruits and vegetables, red and processed meat, fish and shellfish, and waist circumference. Women were additionally matched by menopausal status and phase of menstrual cycle at blood collection; postmenopausal women by hormone replacement therapy use.

[b] *P* values for interaction were calculated with the Wald-test for a multiplicative interaction term of LCN2 and the continuous waist circumference variable.

Table S6. Hazard Ratios (HRs)[a] and 95% confidence intervals (95% CIs) of LCN2 and CRC and subsites, excluding cases (N_{cases} = 77) and matched controls ($N_{controls}$ = 77) with missing covariates, stratified by sex and waist circumference.

**Outcome**	**LCN2 log_2**				$P_{interaction}$[b]
	**Waist circumference**				
	**<94cm (men), <80cm (women)**		**≥94cm (men), ≥80cm (women)**		
	**HR (95% CI)**	***P***	**HR (95% CI)**	***P***	
**Colorectal cancer, *N* (cases/controls)**					
Both sexes	445/527		745/663		
	0.76 (0.68 – 0.85)	<0.01	1.38 (1.27 – 1.49)	<0.01	<0.01
Men	196/248		367/315		
	0.42 (0.35 – 0.51)	<0.01	1.20 (1.09 – 1.33)	<0.01	<0.01
Women	249/279		378/348		
	0.87 (0.76 – 0.99d)	0.04	2.01 (1.75 – 2.30)	<0.01	<0.01
**Colon cancer, *N* (cases/controls)**					
Both sexes	273/330		468/411		
	0.76 (0.67 – 0.88)	<0.01	1.66 (1.50 – 1.84)	<0.01	0.02
Men	111/147		213/177		
	0.25 (0.18 – 0.32)	<0.01	1.52 (1.32 – 1.76)	<0.01	<0.01
Women	162/183		255/234		
	1.04 (0.88 – 1.22)	0.63	3.09 (2.60 – 3.66)	<0.01	<0.01
**Proximal colon cancer, *N* (cases/controls)**					
Both sexes	119/135		198/182		
	0.76 (0.62 – 0.82)	0.01	3.05 (2.58 – 3.59)	<0.01	<0.01
Men	46/59		87/74		
	0.18 (0.11 – 0.29)	<0.01	1.24 (0.97 – 1.59)	0.09	<0.01
Women	73/76		111/108		

	1.15 (0.84 – 1.56)	0.38	6.91 (5.32 – 8.98)	<0.01	<0.01
**Distal colon cancer, *N* (cases/controls)**					
Both sexes	129/177		239/191		
	0.50 (0.39 – 0.64)	<0.01	2.29 (1.94 – 2.72)	<0.01	<0.01
Men	50/77		111/84		
	0.04 (0.02 – 0.07)	<0.01	2.13 (1.62 – 2.78)	<0.01	<0.01
Women	79/100		128/107		
	1.02 (0.73 – 1.42)	0.90	5.64 (4.18 – 7.61)	<0.01	<0.01
**Rectal cancer, *N* (cases/controls)**					
Both sexes	168/194		266/240		
	0.95 (0.78 – 1.15)	0.58	1.18 (1.03 – 1.35)	0.02	0.43
Men	83/99		146/130		
	0.85 (0.63 – 1.15)	0.29	1.15 (0.97 – 1.36)	0.11	0.75
Women	85/95		120/110		
	0.91 (0.68 – 1.22)	0.53	1.00 (0.78 – 1.29)	1.00	0.18

[a] Based on weighted Cox proportional hazard regression using inverse probability weighting to approximate the full cohort to evaluate the association of LCN2 with colorectal cancer risk per doubling of LCN2 concentration. HRs were adjusted for age, sex, study center, follow-up time since blood collection, time of the day at blood collection, fasting status, smoking status, alcohol consumption, physical activity, fiber intake, consumption of fruits and vegetables, red and processed meat, fish and shellfish, and waist circumference. Women were additionally matched by menopausal status and phase of menstrual cycle at blood collection; postmenopausal women by hormone replacement therapy use.

[b] *P* values for interaction were calculated with the Wald-test for a multiplicative interaction term of LCN2 and the continuous waist circumference variable.

Table S7. Hazard Ratios (HRs)[a] and 95% confidence intervals (95% CIs) of LCN2 and CRC and subsites, only including postmenopausal women cases (N_{cases} = 509) and matched controls ($N_{controls}$ = 509), stratified by waist circumference.

**Outcome**	**LCN2 log_2**				$P_{interaction}$[b]
	**Waist circumference**				
	**<80cm**		**≥80cm**		
	**HR (95% CI)**	***P***	**HR (95% CI)**	***P***	
**Colorectal cancer, *N* (cases/controls)**					
Women	201/227		308/282		
	0.81 (0.69 – 0.94)	<0.01	2.18 (1.85 – 2.57)	<0.01	0.12
**Colon cancer, *N* (cases/controls)**					
Women	132/148		216/196		
	0.89 (0.73 – 1.08)	0.23	3.52 (2.88 – 4.32)	<0.01	<0.01
**Proximal colon cancer, *N* (cases/controls)**					
Women	65/69		96/92		
	0.68 (0.46 – 1.01)	0.06	7.09 (5.03 – 9.98)	<0.01	<0.01
**Distal colon cancer, *N* (cases/controls)**					
Women	58/75		107/90		
	0.84 (0.57 – 1.24)	0.39	4.95 (3.48 – 7.05)	<0.01	<0.01
**Rectal cancer, *N* (cases/controls)**					
Women	70/78		91/83		
	1.04 (0.74 – 1.46)	0.84	0.97 (- 0.72 – 1.30)	0.84	0.03

[a] Based on weighted Cox proportional hazard regression using inverse probability weighting to approximate the full cohort to evaluate the association of LCN2 with colorectal cancer risk per doubling of LCN2 concentration. HRs were adjusted for age, study center, follow-up time since blood collection, time of the day at blood collection, fasting status, smoking status, alcohol consumption, physical activity, fiber intake, consumption of fruits and vegetables, red and processed meat, fish and shellfish, and waist circumference. Women were additionally matched by menopausal status and phase of menstrual cycle at blood collection; postmenopausal women by hormone replacement therapy use.

[b] *P* values for interaction were calculated with the Wald-test for a multiplicative interaction term of LCN2 and the continuous waist circumference variable.

Table S8. Multivariable-adjusted hazard ratios [a] (HRs) and 95% confidence intervals (95% CIs) for the association of LCN2 with colorectal cancer comparing high vs. low LCN2 levels based on a-priori defined cut-points.

LCN2 cut-points	≥50 ng/mL vs. <50 ng/mL		≥30 ng/mL vs. <30 ng/mL		≥25 ng/mL vs. <25 ng/mL	
	HR (95%CI)	*P*-value	HR (95%CI)	*P*-value	HR (95%CI)	*P*-value
Both Sexes	1.56 (1.33 - 1.82)	<0.001	1.10 (1.03 - 1.18)	0.005	1.00 (0.94 - 1.06)	0.906
Men	0.78 (0.61 - 0.99)	0.04	0.77 (0.70 - 0.85)	<0.001	0.87 (0.79 - 0.95)	0.002
Women	3.24 (2.55 - 4.13)	<0.001	1.46 (1.33 - 1.61)	<0.001	1.09 (1.00 - 1.18)	0.055

[a] Based on weighted Cox proportional hazard regression using inverse probability weighting to approximate the full cohort to evaluate the association of dichotomized LCN2 values according to predefined cut-points with colorectal cancer risk. HRs were adjusted for age, study center, follow-up time since blood collection, time of the day at blood collection, fasting status, smoking status, alcohol consumption, physical activity, fiber intake, consumption of fruits and vegetables, red and processed meat, fish and shellfish, and waist circumference. Women were additionally matched by menopausal status and phase of menstrual cycle at blood collection; postmenopausal women by hormone replacement therapy use.

Table S9. Multivariable-adjusted hazard ratios [a] (HRs) and 95% confidence intervals (95% CIs) for the association of LCN2 with colorectal cancer comparing high vs. low LCN2 levels according to predefined cu-points, stratified by waist circumference categories.

	Waist circumference categories			
	<94cm (men), <80cm (women)		≥94cm (men), ≥80cm (women)	
	HR (95%CI)	*P*-value	HR (95%CI)	*P*-value
		≥50 ng/mL vs. <50 ng/mL		
Both Sexes	1.11 (0.87 - 1.41)	0.413	2.18 (1.76 - 2.69)	<0.001
Men	0.73 (0.51 - 1.05)	0.092	1.14 (0.82 - 1.58)	0.443
Women	1.21 (0.83 - 1.77)	0.322	9.06 (6.43 - 12.76)	<0.001
		≥30 ng/mL vs. <30 ng/mL		
Both Sexes	0.83 (0.74 - 0.92)	0.001	1.45 (1.33 - 1.59)	<0.001
Men	0.57 (0.49 - 0.68)	<0.001	1.02 (0.90 - 1.16)	0.75
Women	0.84 (0.71 - 0.99)	0.044	2.31 (2.03 - 2.63)	<0.001
		≥25 ng/mL vs. <25 ng/mL		
Both Sexes	0.85 (0.77 - 0.94)	0.001	1.16 (1.07 - 1.25)	<0.001
Men	0.72 (0.62 - 0.83)	<0.001	0.98 (0.87 - 1.10)	0.682
Women	0.79 (0.69 - 0.91)	0.001	1.32 (1.17 - 1.49)	<0.001

[a] Based on weighted Cox proportional hazard regression using inverse probability weighting to approximate the full cohort to evaluate the association of dichotomized LCN2 values according to predefined cut-points with colorectal cancer risk. HRs were adjusted for age, study center, follow-up time since blood collection, time of the day at blood collection, fasting status, smoking status, alcohol consumption, physical activity, fiber intake, consumption of fruits and vegetables, red and processed meat, fish and shellfish, and waist circumference. Women were additionally matched by menopausal status and phase of menstrual cycle at blood collection; postmenopausal women by hormone replacement therapy use.

Supplemental References

1. Eichelmann F, Rudovich N, Pfeiffer AF, et al. Novel adipokines: methodological utility in human obesity research. Int J Obes (Lond). 2017;41(6):976-81. Epub 2017/03/16.
2. Leufkens AM, van Duijnhoven FJ, Woudt SH, et al. Biomarkers of oxidative stress and risk of developing colorectal cancer: a cohort-nested case-control study in the European Prospective Investigation Into Cancer and Nutrition. Am J Epidemiol. 2012;175(7):653-63. Epub 20120315.
3. Aleksandrova K, Boeing H, Jenab M, et al. Total and high-molecular weight adiponectin and risk of colorectal cancer: the European Prospective Investigation into Cancer and Nutrition Study. Carcinogenesis. 2012;33(6):1211-8. Epub 20120319.
4. van Duijnhoven FJ, Bueno-De-Mesquita HB, Calligaro M, et al. Blood lipid and lipoprotein concentrations and colorectal cancer risk in the European Prospective Investigation into Cancer and Nutrition. Gut. 2011;60(8):1094-102. Epub 2011/03/09.
5. Stekhoven DJ, Buhlmann P. MissForest--non-parametric missing value imputation for mixed-type data. Bioinformatics. 2012;28(1):112-8. Epub 2011/11/01.
